# A Linear Fragment of Unacylated Ghrelin (UAG_6−13_) Protects Against Myocardial Ischemia/Reperfusion Injury in Mice in a Growth Hormone Secretagogue Receptor-Independent Manner

**DOI:** 10.3389/fendo.2018.00798

**Published:** 2019-01-11

**Authors:** David N. Huynh, Hanan Elimam, Valérie L. Bessi, Liliane Ménard, Yan Burelle, Riccarda Granata, André C. Carpentier, Huy Ong, Sylvie Marleau

**Affiliations:** ^1^Faculté de pharmacie, Université de Montréal, Montréal, QC, Canada; ^2^Faculty of Pharmacy, University of Sadat City, Sadat, Egypt; ^3^Faculty of Health Sciences, University of Ottawa, Ottawa, ON, Canada; ^4^Department of Medical Science, University of Turin, Turin, Italy; ^5^Division of Endocrinology, Department of Medicine, Université de Sherbrooke, Sherbrooke, QC, Canada

**Keywords:** cardiac ischemia/reperfusion, unacylated ghrelin analogs, hemodynamics, growth hormone secretagogue receptor, tumor necrosis factor-α, interleukin-6, isolated heart

## Abstract

Unacylated ghrelin (UAG), the most abundant form of ghrelin in circulation, has been shown to exert cardioprotective effect in experimental cardiopathies. The present study aimed to investigate the cardioprotective effect of a linear bioactive fragment of UAG against myocardial ischemia-induced injury and dysfunction in C57BL/6 wild type mice and the mechanisms involved. Treatments were administered at doses of 100 (UAG), 1,000 and 3,000 (UAG_6−13_) nmol/kg at 12 h interval during 14 days prior to 30 min left coronary artery ligation and reperfusion for a period of 6 or 48 h. The infarct area was decreased in a dose-dependent manner at 48 h of reperfusion, with a reduction of 54% at the highest dose of UAG_6−13_ tested. Myocardial hemodynamics were improved as demonstrated by an increase in cardiac output, maximum first derivative of left ventricular pressure, and preload recruitable stroke work, a load-independent contractility index. Six hours after reperfusion, circulating levels of IL-6 and TNF-α pro-inflammatory cytokines were reduced, and the effect was maintained at 48 h for TNF-α. 5′ AMP-activated protein kinase (AMPK) was activated, while acetyl-CoA carboxylase (ACC) activity was inhibited, along with a decrease in apoptotic protein levels. In isolated hearts, the effect of UAG_6−13_ was unaffected by the presence of D-Lys^3^-GHRP-6, a ghrelin receptor (GHSR1a) antagonist, suggesting that the peptide acted through a GHSR1a-independent pathway. The results support the therapeutic application of UAG bioactive peptide fragments against myocardial ischemia/reperfusion injury.

## Introduction

Ghrelin, an endogenous selective ligand of growth hormone secretagogue receptor (GHSR), was discovered by Kojima et al. as the first known circulating octanoylated peptide (1). The predominant circulating form of ghrelin in circulation is unacylated in position 3 (unacylated ghrelin–UAG) (2). In contrast to acylated ghrelin (AG), UAG is devoid of growth hormone (GH)-releasing activity, orexigenic, adipogenic and diabetogenic activities; however it shares cardioprotective effects mediated through a still elusive receptor independent of GHSR1a (3). Although the mechanism through which UAG exerts cardioprotection has not been fully elucidated, activation of pro-survival pathways, in addition to anti-inflammatory and antifibrotic activities [reviewed in (4)], supports its therapeutic potential in myocardial ischemia/reperfusion (MI/R) injury. Indeed, MI/R is associated with intense inflammation, apoptosis, and ensuing morbidity associated with pathological remodeling (5, 6).

Structure-activity relationship studies of UAG uncovered the bioactivity of shorter fragments devoid of serine in the third position (7). In particular, linear UAG_6−13_ (Ser-Pro-Glu-His-Gln-Arg-Val-Gln-NH_2_) and its head-to-tail cyclic derivative (cUAG_6−13_), retain UAG bioactivity in enhancing survival of pancreatic islets and beta cells and diminishing oxidative stress and senescence of human circulating endothelial progenitor cells (7). UAG_6−13_ blocked streptozotocin-induced diabetes in rats (7), whereas cUAG_6−13_ (AZP-531) reduced high fat diet-associated inflammation and prediabetes in mice (8). Administration of AZP-531 in mice subjected to transient left coronary artery ligation (LCAL) at 5 min (pre-) and 30 min (post-) reperfusion reduced myocardial infarction through attenuating reperfusion-elicited oxidative stress and activating pro-survival pathways (9).

The present study aims to investigate the cardioprotective effect of linear UAG_6−13_ in reducing MI/R-induced injury and improving hemodynamic performance and to examine underlying mechanisms.

## Materials and Methods

### Mice Treatments and Surgery

All experimental procedures were approved by the Institutional Animal Ethics Committee, in accordance with the Canadian Council on Animal Care guidelines for use of experimental animals and to the Guide for the Care and Use of Laboratory Animals published by the US National Institutes of Health (A5213-01).

Four-month-old male C57BL/6 mice, bred and maintained in-house, were used to determine the effect of UAG_6−13_ pretreatment on myocardial infarct size and myocardial functional recovery after 30 min of ischemia and 48 h of reperfusion. UAG-treated mice were used as a positive control group with known cardioprotective effects. Additional groups of mice were used to investigate the effect of UAG_6−13_ pretreatment on inflammatory and/or metabolic critical processes in the early (6 h) post-reperfusion stage. The isolated perfused heart model was used to determine whether myocardial GHSR1a was involved in the cardioprotective effect of UAG_6−13_ fragment through pharmacological means, e.g., use of AG receptor antagonist (D-Lys^3^-GHRP-6).

**Figure 1:**
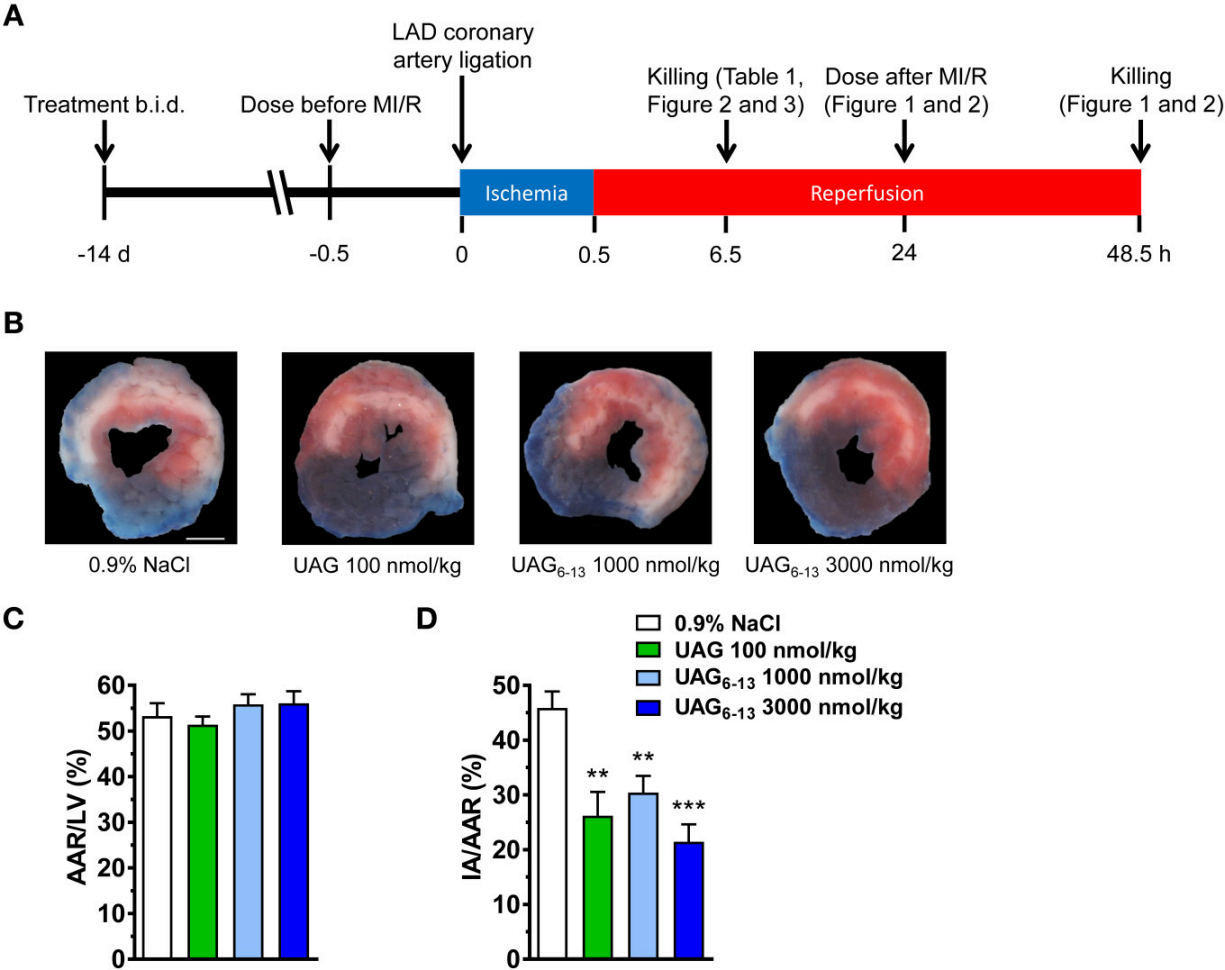
UAG_6−13_ reduced infarct area following MI/R. **(A)** Schematic representation of the experimental protocols. **(B)** Representative photomicrographs of Evans Blue and triphenyltetrazolium chloride (TTC) double stained midventricular LV slices showing the IA on the anterior section. **(C)** Bar graphs of % AAR/LV and **(D)** IA/AAR after a 2-week pretreatment every 12 h with the last dose administered 30 min before myocardial ligation and 48 h reperfusion. Data are mean ± SEM of *n* = 6 mice per group. ^**^P < 0.01 and ^***^P < 0.001 vs. vehicle. Scale bar, 1 mm.

MI/R was performed as described previously (10, 11), as shown in Figure 1A. Briefly, C57BL/6 mice were pretreated during 14 days by twice daily subcutaneous (s.c.) injections of UAG (100 nmol/kg), UAG_6−13_ (1 or 3 μmol/kg), or vehicle (0.9% NaCl). The last dose of these drugs was administered 30 min prior to ligation of the left anterior descending (LAD) coronary artery and on the following day, where applicable. Mice were injected intraperitoneally (i.p.) with buprenorphine (0.05 mg/kg) prior to endotracheal intubation and ventilated using Minivent mouse ventilator (Harvard Apparatus, Holliston, MA, USA) during surgery under isoflurane anesthesia. Transient myocardial ischemia was induced by the insertion of an 8–0 nylon suture tied over a piece of tubing at 1 mm below the edge of atrial appendage. Lidocaine (6 mg/kg) i.p. was injected just after occlusion and 10 min prior to reperfusion to prevent fatal arrhythmias. The chest cavity was closed using a 6–0 silk suture and the animals were allowed to recover for 6 or 48 h. Mice subjected to 6 h of reperfusion were anesthetized with isoflurane and euthanized by exsanguination. Assessment of the impact of treatments on myocardial infarction area was performed following re-occlusion of the original ligation site after 48 h of reperfusion, followed by injecting 300 μL of KCl (1 M) in the subclavian vein and Evans blue (2%) through the aorta. Analysis was performed on triphenyltetrazolium chloride (TTC) (1%)-stained slices by computerized planimetry by an observer blinded to the treatment protocol (10).

### Myocardial Hemodynamics as Assessed by Conductance Catheter

Left ventricle (LV) function parameters were derived from pressure-volume (PV) analysis using a miniaturized PV conductance catheter, as described previously (10). Briefly, a left thoracotomy was performed at 48 h post-surgery, and a microtip 1.4 Fr PV catheter (SPR 839, Millar Instruments) was inserted through the LV apex, connected to a transducer system (Millar). PV signals were recorded at steady-state and during vena cava occlusion to reduce pre-load transiently. Absolute volume data were adjusted by inserting the catheter in a cuvette with pre-defined volume containing heparinized blood at 37°C at the end of each experiment and corrected for the parallel conductance through the hypertonic saline bolus injection method. Data were analyzed using IOX2 software (EMKA technologies, Paris, France).

### Isolated Perfused Heart Assay

Hearts from anesthetized (3% isoflurane mixed with 100% oxygen) and heparinized (5,000 U/kg, i.p.) male C57BL/6 mice were isolated and immersed in ice-cold Krebs-Henseleit bicarbonate buffer containing 118 mM NaCl, 25 mM NaHCO_3_, 4.7 mM KCl, 1.2 mM KH_2_PO_4_, 2.5 mM CaCl_2_, 1.2 mM MgSO_4_, 11 mM glucose, 8 nM insulin, 1.5 mM lactate, 0.2 mM pyruvate, and 0.5 mM EDTA, as described previously (11). After pericardial fat removal, the aorta was cannulated with an 18-gauge cannula, and the heart was perfused retrogradely in a non-recirculating mode at a constant perfusion pressure of 80 mmHg with Krebs-Henseleit bicarbonate buffer supplemented with 1.2 mM palmitate in 2% fatty acid-free bovine serum albumin and gassed with 95% O_2_-5% CO_2_. Hearts were subjected to 30 min of aerobic perfusion, followed by 40 min of low-flow ischemia (10% of preischemic flow) and 10 min of reperfusion. Hearts with rate of perfusion pressure (RPP) <10,000 mmHg/min during baseline were excluded from the study. AG, D-Lys^3^-GHRP-6 and UAG_6−13_ were administered in a non-recirculating mode via retrograde perfusion throughout the 10 min of reperfusion post-ischemia via a side port directly above the aortic cannula at final concentrations of 1, 10, and 30 μM, respectively. Contractile function was monitored continuously by means of a fluid-filled balloon inserted in the LV connected to a pressure transducer. Ventricular pressures and heart rates were recorded with a PowerLab/8sp instrument and analyzed with Chart v5.0.1 software (AD Instruments, Bella Vista, NSW, Australia). Coronary effluent was collected at various time intervals for measurements of coronary flow.

### Western Blots

LV were homogenized using a dounce homogenizer (TRI-R S63C, ON, Canada) in PBS containing a protease and phosphatase inhibitors cocktail (Thermo Scientific Pierce). Homogenates were separated into two tubes. Total homogenates were obtained by sonication for 60 min on ice with an equal volume of lysis buffer (150 mM NaCl, 100 mM Tris-HCl, 2% Triton X-100, 0.2% SDS, pH = 7.4), centrifuged at 12,000 *g* for 20 min at 4°C, and the supernatant was kept at −80°C. To collect the cytosolic fraction, the other tube was centrifuged at 1,000 *g* for 10 min at 4°C, the supernatant was centrifuged at 9,000 *g* for 10 min at 4°C, and the supernatant was kept at −80°C. The protein concentration was determined by the BCA assay (Pierce Biotechnology). Equal amounts (25 μg) of protein extracts were loaded on SDS-polyacrylamide gels and transferred to PVDF membranes (Bio-Rad Laboratories). After blocking, membranes were incubated overnight at 4°C with the following primary antibodies; polyclonal rabbit anti-Akt and anti-phosphoSer473-Akt were used for the total homogenates, while polyclonal rabbit anti-acetyl-coenzyme A carboxylase (ACC), anti-phosphoSer79-ACC (ACC1 and ACC2), anti-5′ AMP-activated protein kinase (AMPK)α1/α2, anti-phosphoThr172-AMPK (1:1,000 Cell Signaling Technology, Danvers, MA, USA), monoclonal mouse anti-cytochrome c (BD Biosciences, Franklin Lakes, NJ, USA), polyclonal goat anti-AIF (Santa Cruz, Dallas, TX, USA) were used for the cytosolic fractions. Monoclonal mouse anti-α-tubulin (1:5,000, Abcam) was used as an internal control and detected on the same membrane (Supplemental Figures 2–10). Membranes were incubated with HRP-conjugated secondary goat anti-rabbit IgG (1:10,000, Jackson Immunoresearch), goat anti-mouse IgG (1:10,000, KPL) or donkey anti-goat IgG (1:10,000, Abcam). Bands from the same blot were detected by enhanced chemiluminescence and analyzed using ChemiDoc XRS+ (Bio-Rad, ON, Canada).

### Plasma Cytokines

Blood from non-fasted mice was collected on 4 mM EDTA to determine interleukin-6 (IL-6) and tumor necrosis factor-α (TNF-α) plasma levels using commercial kits (eBioscience, Waltham, MA, USA; catalog numbers 88-7064 and 88-7324, respectively) by following manufacturer's instructions.

### Statistical Analysis

Data are represented as mean ± SEM. Comparisons between groups were performed using unpaired one- or two-way ANOVA followed by Student-Newman-Keuls (SNK) method for multiple *post hoc* comparisons (GraphPad Prism Version 7.02, San Diego, CA). Probability values <0.05 were considered statistically significant.

## Results

### UAG and UAG_6−13_ Pretreatments Protect From Myocardial Ischemia/Reperfusion Injury and Preserve LV Hemodynamics

C57BL/6 mice were pretreated with UAG, UAG_6−13_ or 0.9% NaCl (vehicle) at 12 h interval for 14 days and 30 min prior to LCAL induced by the proximal occlusion of the LAD coronary artery, followed by 6 or 48 h of reperfusion (Figure 1A). Our results display a consistently large myocardial area-at-risk (AAR) (slightly more than 50% of the LV area) in all groups as shown by representative photomicrographs of TTC-stained midventricular, transverse slices (Figure 1B). The AAR/LV ratio did not differ significantly between groups (Figure 1C), suggesting that the ligation was reproducibly performed at the same LAD level. We first investigated the cardioprotective effect of UAG at a dose equimolar to that previously published for AG shown to exert a cardioprotective effect in experimental cardiopathies (12, 13). Our results show that prophylactic administration of UAG (100 nmol/kg twice daily), used as a positive control, significantly reduced infarct area (IA) to AAR by 43%, whereas 10–30-fold higher doses of UAG_6−13_ reduced myocardial infarction to a similar extent (34–54%) (Figure 1D).

In parallel studies, the effect of a preventive administration of UAG and UAG_6−13_ on systemic hemodynamics and indices of systolic and diastolic function was determined using conductance catheter-derived PV relationship at 48 h of reperfusion as carried out previously (10, 11). Vehicle-treated mice exhibited similar LV dysfunction as those subjected to the same MI/R protocol (10, 11). Compared to vehicle-treated mice, UAG_6−13_ improved systemic hemodynamics by increasing stroke volume and cardiac output by 40 and 58%, respectively (Table 1). Although systolic LV indices, including ejection fraction (EF), end systolic pressure (ESP), and end systolic volume (ESV), were modestly improved by the peptides at their respective administered doses, stroke work was increased by 55 and 81% by UAG and UAG_6−13_, respectively. The peak rate of rise in the first derivative of left ventricular pressure (dP/dt_max_) was increased by ~40% by both treatments as well as that of preload recruitable stroke work (PRSW), a load-independent indice of contractility, by 49% (UAG) and 39% (UAG_6−13_). Reduced tau and increased dP/dt_min_ suggested improved diastolic relaxation.

**Table 1:** Hemodynamic measurements at 6 h after myocardial ischemia caused by a 30 min coronary artery ligation in mice.

	**Treatment**		
**Parameter**	**0.9% NaCl**	**UAG 100 nmol/kg**	**UAG_**6−13**_ 3,000 nmol/kg**
BW, g	24.9 ± 0.7	25.5 ± 1.3	25.4 ± 0.7
HR, min^−1^	400 ± 15	410 ± 15	436 ± 15
SV, μL	10 ± 1	12 ± 1	14 ± 1^*^
CO, mL/min	4.0 ± 0.4	5.2 ± 0.5	6.3 ± 0.4^**^
Ea, mmHg/μL	6.3 ± 0.5	6.2 ± 0.6	5.3 ± 0.4
SVR, mmHg min/mL	16 ± 2	16 ± 2	13 ± 1
**SYSTOLIC INDICES**			
EF, %	64 ± 4	77 ± 3^**^	82 ± 2^**^
ESP, mmHg	61 ± 3	75 ± 5^**^	76 ± 3^*^
ESV, μL	6.5 ± 0.3	4.7 ± 0.3^***^	4.5 ± 0.3^***^
dP/dt max, mmHg/s	*4, 408*±227	*5, 885*±463**	*6, 517*±313^***^
SW, mmHg μL	505 ± 56	781 ± 82^*^	916 ± 81^**^
PRSW, mmHg	49 ± 2	73 ± 5^***^	68 ± 4^**^
Ees, mmHg/μL	8.1 ± 1.3	12.0 ± 2.2	10.2 ± 1.6
Ea/Ees	0.89 ± 0.09	0.57 ± 0.05^*^	0.61 ± 0.09^*^
**DIASTOLIC INDICES**			
EDP, mmHg	3.8 ± 0.4	3.9 ± 0.4	3.8 ± 0.6
EDV, μL	14.9 ± 0.5	15.2 ± 0.9	16.8 ± 0.8
dP/dt min, mmHg/s	−3,725 ± 218	−5,027 ± 471^**^	−5,479 ± 266^**^
EDPVR, mmHg/μL	0.35 ± 0.04	0.39 ± 0.04	0.35 ± 0.05
tau, ms	13.3 0.7	11.5 ± 1.2	10.0 ± 0.6^*^

*Mice were randomized to one of three study groups (n = 9-11 per group) and treated twice per day at 12 h interval. BW, body weight; HR, heart rate; SV, stroke volume; CO, cardiac output; Ea, arterial elastance; Ees, end-systolic elastance; SVR, systemic vascular resistance; EF, ejection fraction; ESP, end systolic pressure; ESV, end systolic volume; dP/dt max (or min), maximal rate of pressure increase (or decline); SW, stroke work; PRSW, preload recruitable stroke work; EDP, end diastolic pressure; EDV, end diastolic volume; EDPVR, end-diastolic pressure-volume relationship; tau, time constant of relaxation (varying asymptote method). Data are mean ± SEM. One-way ANOVA was applied for comparisons with the Student-Newman-Keuls post hoc comparisons*.

* *P < 0.05*,

** P < 0.01 and

*** *P < 0.001 vs. vehicle-treated mice*.

### UAG and UAG_6−13_ Pretreatments Exert Anti-Inflammatory Effects

**Figure 2:**
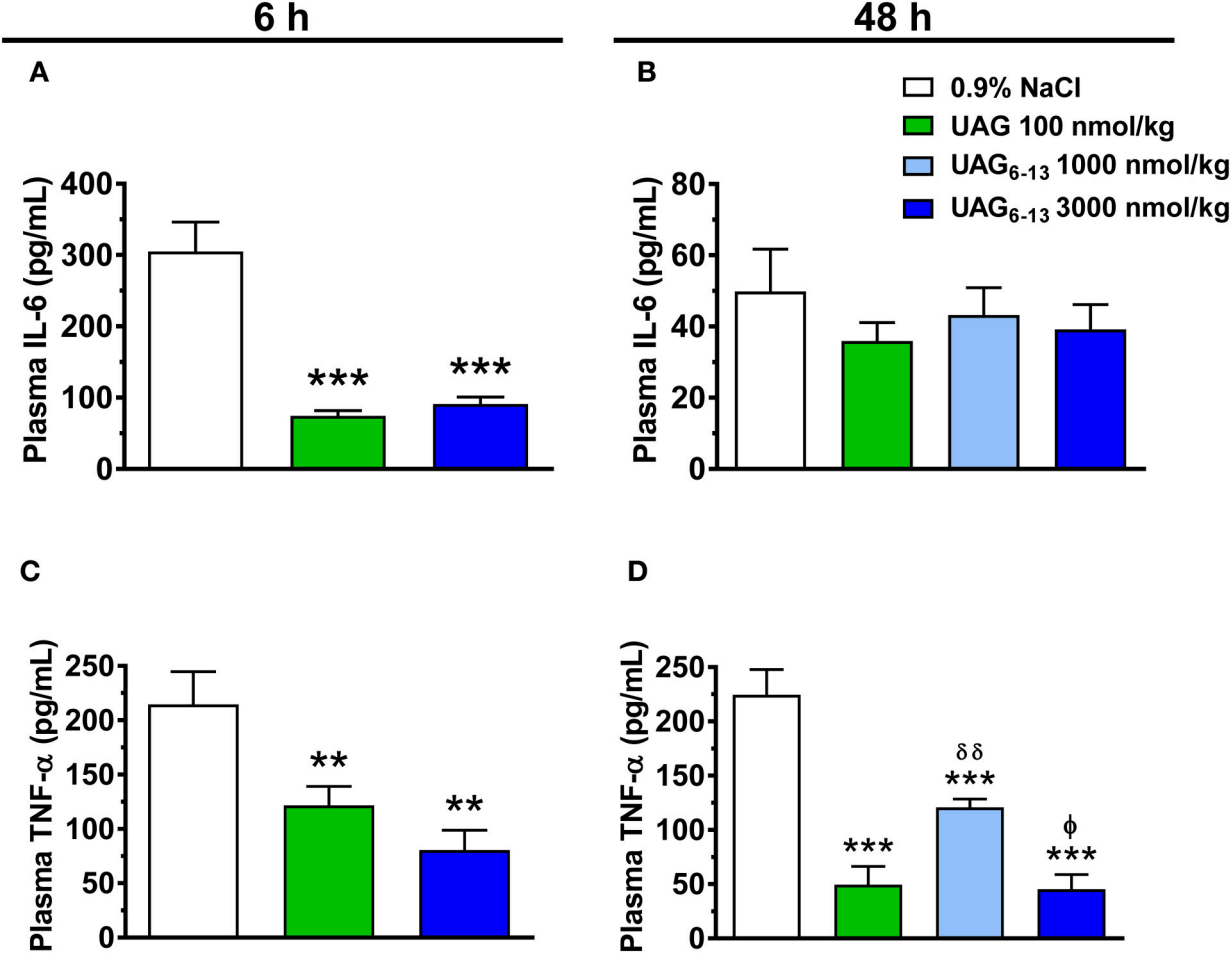
UAG_6−13_ reduced inflammation following MI/R. Bar graphs represent plasma IL-6 concentration at **(A)** 6 h and **(B)** 48 h of reperfusion following a 30-min LAD ligation. Bar graphs show TNF-α plasma concentration at **(C)** 6 h and **(D)** 48 h of reperfusion. Data are mean ± SEM of *n* = 9–11 mice (6 h time point) and *n* = 6 mice (48 h) per group. ^**^P < 0.01 and ^***^P < 0.001 vs. vehicle, ^δδ^*P* < 0.01 vs. UAG 100 nmol/kg and ^ϕ^*P* < 0.05 vs. UAG_6−13_ 1,000 nmol/kg.

We investigated the potential anti-inflammatory effect of UAG and UAG_6−13_ peptides in early (6 h) and late (48 h) reperfusion following myocardial ligation. Our results show that after 14 d pretreatment twice daily, UAG (100 nmol/kg) and UAG_6−13_ (3,000 nmol/kg) transiently reduced plasma IL-6 levels by 76 and 70% at 6 h, respectively, whereas this effect vanished by 48 h (Figures 2A,B). In a similar manner, substantial inhibitory effect of treatments was observed on plasma TNF-α levels at 6 h following MI/R, by 43% (UAG) and 62% (UAG_6−13_). The inhibitory effect was maintained at 48 h, as shown by a 78% in UAG-treated, and 46 and 80% reductions in UAG_6−13_ (1,000 and 3,000 nmol/kg)-treated mice (Figures 2C,D).

Neither UAG nor UAG_6−13_ pretreatment modulated body weight, food or water intake, glycemia, insulin and non-esterified fatty acid (NEFA) plasma levels (Supplemental Figure 1).

### Phosphorylation of Myocardial AMPK, ACC and Apoptosis-Related Markers After MI/R in UAG and UAG_6−13_-Treated Mice

**Figure 3:**
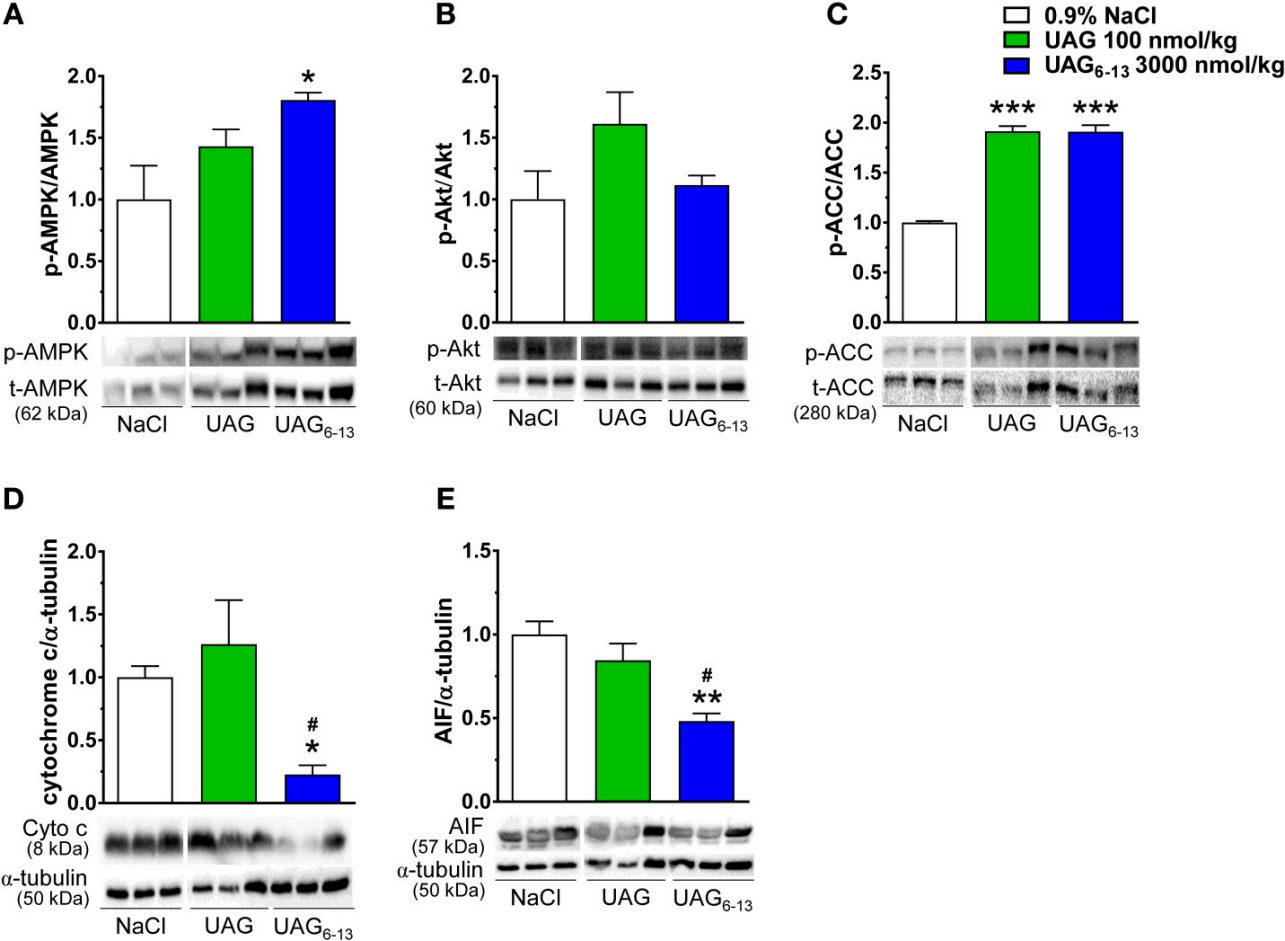
UAG_6−13_ improved cardiac metabolism and reduced apoptosis after MI/R. Bar graphs represent the relative phosphorylated to total protein ratios with representative immunoblots. **(A)** pThr172–AMPKα1/α2 (p-AMPK) to total AMPKα1/α2 ratios and immunoblots from cytosolic LV fractions. **(B)** pSer473-Akt (p-Akt) to total Akt ratios and immunoblots from total LV homogenates. **(C)** pSer79-ACC (p-ACC) and total ACC ratios and immunoblots from cytosolic LV fractions. **(D)** Cytochrome c (Cyto c) and α-tubulin ratios and immunoblots from cytosolic LV fractions. **(E)** AIF and α-tubulin ratios and immunoblots from cytosolic LV fractions. Relative band density ratios were normalized to the vehicle-treated group. Data are mean ± SEM of *n* = 3 mice per treatment group. ^*^P < 0.05, ^**^P < 0.01, and ^***^P < 0.001 vs. vehicle, ^#^*P* < 0.05 vs. UAG 100 nmol/kg.

Western blots analysis of LV protein homogenates showed that the mean relative ratio of pThr172-AMPK to total AMPK band density was increased by 83% after 6 h reperfusion in ischemic hearts of mice treated twice daily with UAG_6−13_ (3,000 nmol/kg), compared with vehicle-treated mice (Figure 3A). In contrast, pSer473-Akt to total mean Akt ratio was unchanged compared to that of vehicle-treated mice at this time-point (Figure 3B). However, the mean ratio of pSer79-ACC to total ACC was increased by 91% in both UAG (100 nmol/kg) and UAG_6−13_ (3,000 nmol/kg)-twice daily treated mice (Figure 3C). Expression of mitochondrial signaling proteins of apoptosis, cytochrome c, and apoptosis inducing factor (AIF), was significantly reduced in LV cytosolic fraction by UAG_6−13_ treatment but not in UAG-treated mice (Figures 3D,E). Complete scanned gels for western blots are shown in Supplemental Figures 2–10.

### UAG_6−13_ Improves Myocardial Hemodynamics in Isolated Hearts in a GHSR1a-Independent Manner

**Figure 4:**
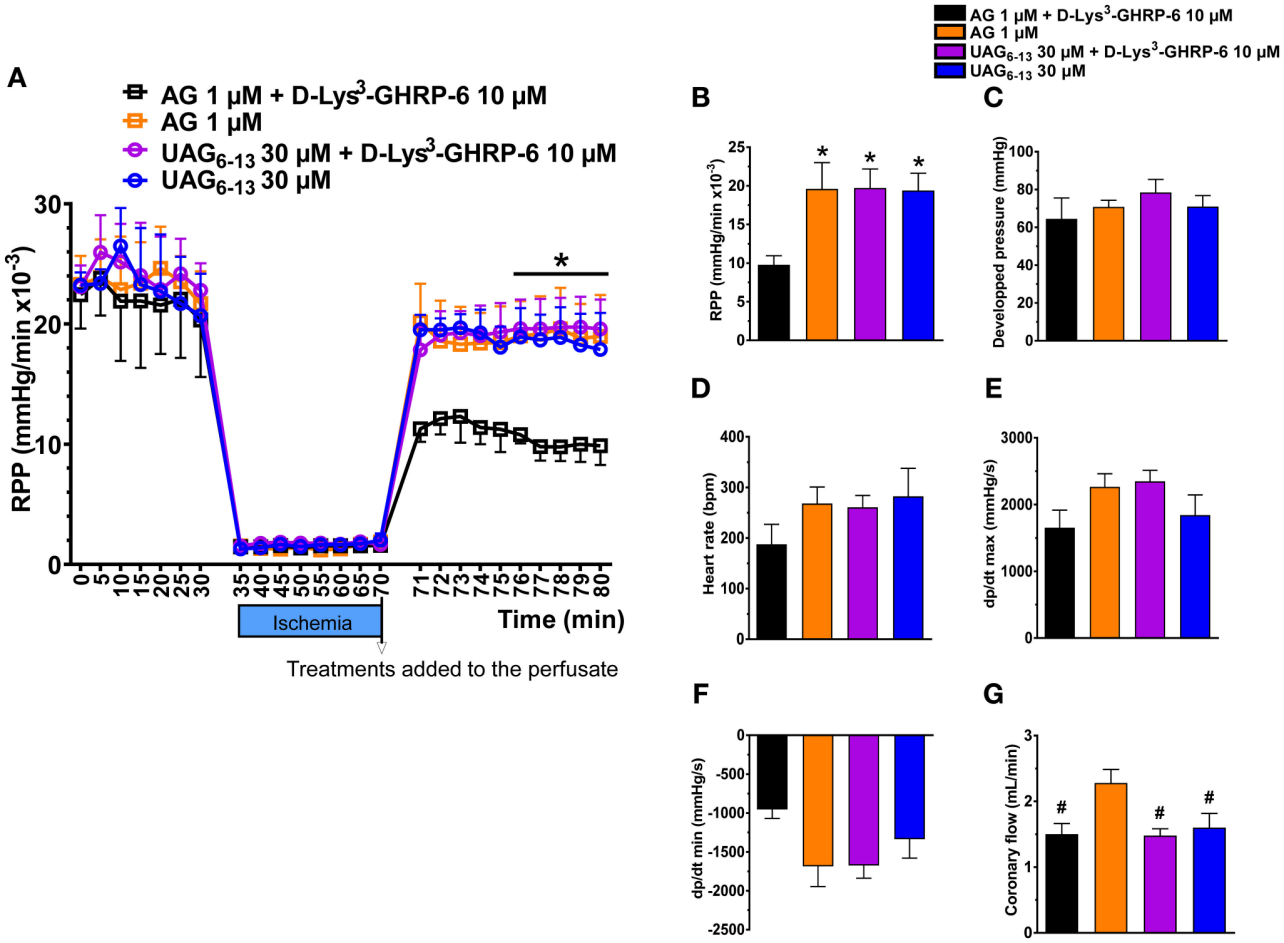
UAG_6−13_ preserved cardiac function in isolated hearts subjected to 40 min of low-flow ischemia and reperfusion in a GHSR1a-independent manner. **(A)** LV rate-pressure product (RPP) before, during and after reperfusion in the presence of either 1 μM AG or 30 μM UAG_6−13_ with or without 10 μM D-Lys^3^-GHRP-6. Data are mean ± SEM of n = 4–5 hearts per group. A two-way ANOVA was performed, followed by Student-Newman-Keuls' *post hoc* comparisons. There was no interaction between time and treatment. ^*^P < 0.05 vs. AG + D-Lys^3^-GHRP-6, by two-way ANOVA followed by Student-Newman-Keuls' *post hoc* comparisons, **(B–G)** Bar graphs of cardiac hemodynamic parameters at 10 min post-ischemia: **(B)** RPP, **(C)** left ventricular–developed pressure, **(D)** heart rate, **(E)** dp/dt max, **(F)** dp/dt min, and **(G)** coronary flow. ^*^P < 0.05 vs. AG + D-Lys^3^-GHRP-6, by one-way ANOVA followed by Student-Newman-Keuls' *post hoc* comparisons, ^#^*P* < 0.05 vs. AG by one-way ANOVA followed by Student-Newman-Keuls' *post hoc* comparisons.

To investigate whether UAG_6−13_ exerts cardioprotective effect through a GHSR1a-independent pathway in the heart, we used an isolated heart model of low-flow ischemia and reperfusion in Langendorff mode to assess the rate of perfusion pressure (RPP) and additional hemodynamic parameters. Hearts from 20 mice were sorted according to the following pharmacological treatments: AG and AG + D-Lys^3^-GHRP-6, as positive and negative control groups; UAG_6−13_ and UAG_6−13_ + D-Lys^3^-GHRP-6, as tested groups. Our results show that the RPP was largely preserved in the presence of both AG and UAG_6−13_ at reperfusion (Figure 4A). Treatment with D-Lys^3^-GHRP-6 completely abolished RPP rescue by AG at reperfusion; in contrast, D-Lys^3^-GHRP-6 did not have any effect on UAG_6−13_-elicited cardioprotection. Consistent with improved RPP in the presence of UAG_6−13_, myocardial hemodynamics tended to be improved by UAG_6−13_ treatment with or without D-Lys^3^-GHRP-6, although no change was observed in heart rate and coronary flow (Figures 4B–G).

## Discussion

The main finding of this study is that a preventive treatment with a linear UAG fragment, UAG_6−13_, reduced MI/R-induced injury and rescued hemodynamic dysfunction in mice, in a GHSR1a-independent manner. The cardioprotective effects were associated with potent, time-dependent, and systemic anti-inflammatory effects of the treatment. These observations are consistent with those of Harisseh et al. who showed that an acute administration of cyclic UAG_6−13_ (cUAG_6−13_ or AZP-531), in a similar dose range, reduced infarct size following MI/R in mice, as a consequence of an early activation of pro-survival and anti-oxidative pathways (9).

AG/GHSR1a axis has been proposed as a promising therapeutic target in a variety of metabolic disorders due to its pleiotropic effects on appetite, energy metabolism, glucose homeostasis, and GH release. While the two circulating forms of ghrelin present distinct endocrine activities (14), they also share many peripheral biological activities, through the binding of an as of yet elusive receptor (15–18). Both forms may be beneficial to rescue myocardial dysfunction elicited by drugs such as isoproterenol or doxorubicin or ischemia in isolated hearts, cardiomyocytes and in preclinical models (12, 13, 19–23). While AG is cardioprotective, its pleiotropic effects and GH secretagogue activity may jeopardize its favorable effects in protecting the heart following long-term exposure with the potential of eliciting serious adverse events such as cancer growth and metastasis (24). In contrast, UAG, the most abundant circulating form of ghrelin, shows GH- and GHSR1a-independent effects in promoting anti-apoptotic pathways in cardiomyocytes and protecting against drug-induced cardiac dysfunction and fibrosis (13).

UAG fragments were studied for their bioactivity and ability to mimic UAG pro-survival effect on beta cells and human pancreatic islets (7). UAG fragment 6-13 showed cardioprotective effect (9), and full cyclization of the peptide did not affect its bioactivity and improved its pharmacokinetics (25). Cyclic UAG_6−13_ improved mitochondrial function, reduced apoptosis and myocardial damage at 15 min and 24 h following reperfusion, respectively. In agreement, our results show reduced myocardial damage at 48 h following reperfusion and further show that these beneficial effects were associated with improved hemodynamics and reduced plasma TNF-α levels at this time point. In addition, the anti-apoptotic effect observed by Harisseh et al. (9) after 15 min of reperfusion appeared to be maintained at 6 h, a time point at which changes in myocardial metabolism is apparent (10). Although the present work does not allow direct comparison of the cardioprotective efficacy of cyclic and linear forms of UAG_6−13_ as experimental designs, doses and endpoints differed between studies, the present work confirms that the UAG_6−13_ fragment is cardioprotective and furthermore, that it also improves cardiac hemodynamics and contractility, as shown by a significant increase in cardiac output, dP/dt_max_ and PRSW.

Both oxidative stress and pro-inflammatory cytokines play a role in promoting an inflammatory response to MI/R (26). In the present study, we further investigated the effect of UAG and UAG_6−13_ on plasma levels of IL-6 and TNF-α at 6 h of reperfusion, a time point at which myocardial levels of these cytokines are elevated in humans (27). Our results show that treatment with UAG and UAG_6−13_ elicited a striking, but transient, reduction of IL-6 plasma levels, whereas TNF-α levels were diminished at both 6 and 48 h after reperfusion. Of interest, UAG and UAG_6−13_ shared a similar transient inhibitory profile for IL-6 and a prolonged effect on TNF-α, notwithstanding differences in doses. The reason for the prolonged effect of the treatment on TNF-α vs. IL-6 plasma levels is not clear, but might be related to the fact that TNF-α clearance is four times lower than IL-6 in rats (28). Elevated levels of circulating inflammatory cytokines, including TNF-α and IL-6, have been associated with myocardial dysfunction and increased apoptosis following MI/R (29). Whereas an acute administration of cUAG_6−13_ (5 min before reperfusion) was shown to increase myocardial anti-oxidative and anti-apoptotic pathways at 15 min after reperfusion (9), a pretreatment with linear UAG_6−13_ (with the last dose administered 30 min before ligation), but not UAG, reduced cytochrome c and AIF protein expression after 6 h of reperfusion, suggesting that both caspase dependent and independent pathways have been activated (30). Our results differ from those of Pei et al. (13) who showed that UAG, in a similar dose range, administered after doxorubicin injection in younger (10–12 weeks old) C57BL/6 mice, showed reduced myocardial apoptosis in treated mice. Yet, it has been reported that doxorubicin-induced cardiac injury promotes endogenous ghrelin release, which may thus contribute to the cardioprotective and anti-apoptotic mechanisms elicited by UAG in that model (31).

As reported previously for HL-1 cardiomyocytes (22), the ratio of p-AMPK/AMPK was not significantly changed in UAG-treated mice, but was increased by UAG_6−13_ at 6 h of reperfusion, which may contribute to protect the heart through the attenuation of TNF-α-elicited apoptosis (32) and/or modulate myocardial energy metabolism. Along these lines, p-ACC/ACC was increased by the pretreatments, leading to enhanced β-oxidation and ATP production (33). Whether myocardial glucose metabolism was increased and fat metabolism decreased by the treatments remain to be investigated.

An enigmatic issue about the receptor or receptor subtypes involved in mediating the effects of UAG and UAG fragments still remains. In the heart, a number of different receptor subtypes have been proposed, including ghrelin receptor-like receptor (GRLR) that would bind ghrelin and UAG as well as UAG receptors and GHSR1a (34). To investigate whether UAG_6−13_ exerted its cardioprotective effect via the ghrelin receptor GHSR1a, we perfused isolated mice hearts with the ghrelin fragment in the presence or absence of the ghrelin antagonist, D-Lys^3^-GHRP-6, a selective antagonist of the AG receptor. Our results showed that while D-Lys^3^-GHRP-6 inhibited AG-induced rescue of RPP, it did not interfere with the effect of UAG_6−13_. These observations support that the cardioprotective effect on myocardial hemodynamics afforded by UAG_6−13_, even at a dose of 30 μM, is likely to be mediated by UAG receptors. In the present study, the effect of UAG_6−13_ was investigated in pretreated mice. Although this experimental design may limit translation to acute clinical situation of MI/R, previous studies investigating the cardioprotective effect of hexarelin, a small synthetic growth hormone peptide, required a 7–14 days pretreatment in rats (35). Yet, hexarelin was later shown to improve cardiac performance by increasing left ventricular ejection fraction in humans following an acute administration (36, 37). The cardioprotective effect of a single dose of the linear octapeptide UAG fragment in mice after the onset of myocardial ischemia remains to be investigated.

In conclusion, the results of this study show that a pretreatment with a linear bioactive fragment of UAG reduced myocardial damage following MI/R and for the first time shows that these beneficial effects are associated with improved myocardial hemodynamics, along with potent and prolonged anti-inflammatory effects over 48 h. These observations add to the early (15 min) anti-oxidative and pro-survival activation pathways seen with the cyclic peptide (9). Furthermore, we show that UAG_6−13_ exerted cardioprotective effects via mechanisms that are independent of GHSR1a. Taken together, these results support further studies to investigate the mechanisms and pharmacokinetics of the peptide for the translation of these observations.

## Author Contributions

DH, HE, VB, and LM performed the experiments and/or assays and analysis. AC, HO, SM, and YB designed the study and analyzed the data. DH, HE, and SM wrote the paper. AC, YB, HO, and RG performed critical reading of the manuscript. All authors contributed to manuscript revision and approved the submitted version.

### Conflict of Interest Statement

The authors declare that the research was conducted in the absence of any commercial or financial relationships that could be construed as a potential conflict of interest.
