# Supplementary material for: A Linear Fragment of Unacylated Ghrelin (UAG6−13) Protects Against Myocardial Ischemia/Reperfusion Injury in Mice in a Growth Hormone Secretagogue Receptor-Independent Manner
Source: Front Endocrinol (Lausanne). 2019 Jan 11;9:798. doi: 10.3389/fendo.2018.00798 (PMC6340090; doi:10.3389/fendo.2018.00798)
Supplement: Supplementary file 1 [file Data_Sheet_1.pdf]

ONLINE DATA SUPPLEMENT

A Linear Fragment of Unacylated Ghrelin (UAG_{6-13}) Protects Against Myocardial Ischemia/Reperfusion Injury in Mice in a Growth Hormone Secretagogue Receptor-Independent Manner

David N. Huynh[1], Hanan Elimam[1,2], Valérie L. Bessi[1], Liliane Ménard[1], Yan Burelle[3], Riccarda Granata[4], André C. Carpentier[5], Huy Ong[1*], and Sylvie Marleau[1*]

[1]Faculté de pharmacie, Université de Montréal, Montréal, Québec, Canada

[2]Faculty of Pharmacy, University of Sadat City, Sadat City, Egypt

[3]Faculty of Health Sciences, University of Ottawa, Ontario, Canada

[4]Department of Medical Science, University of Turin, Turin, Italy

[5]Department of Medicine, Division of Endocrinology, Université de Sherbrooke, Québec, Canada

*Correspondence:
sylvie.marleau@umontreal.ca

huy.ong@umontreal.ca

Supplemental Figure

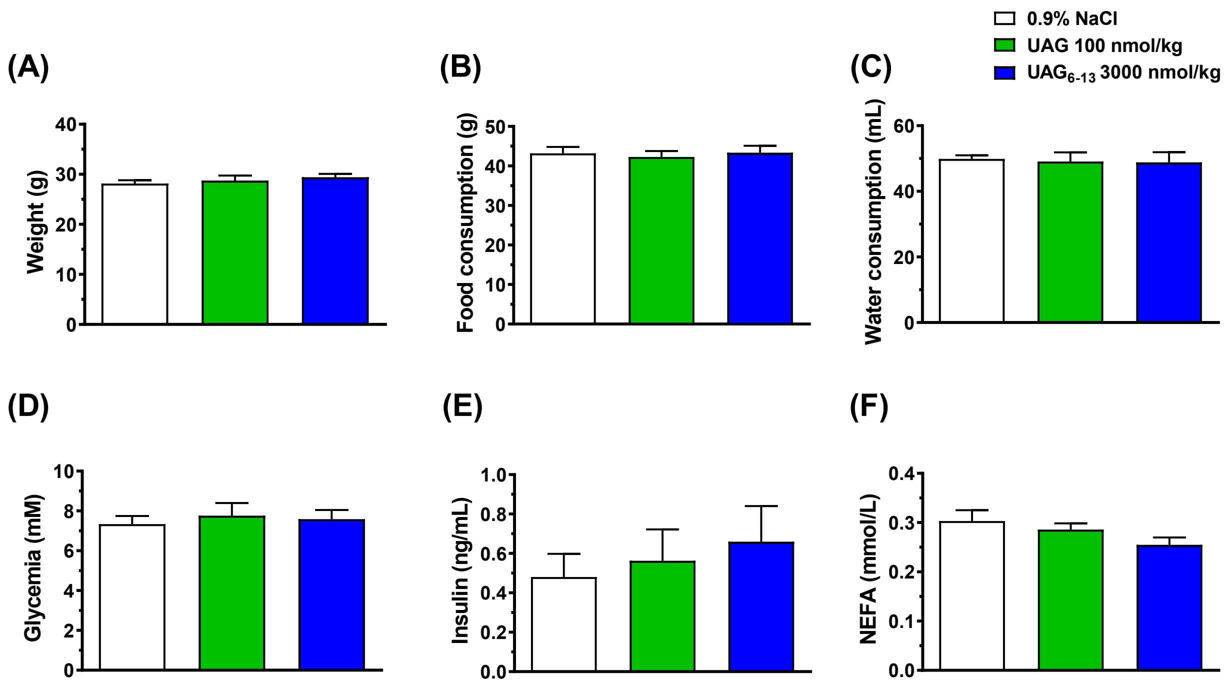

Supplemental Figure 1. Biochemical parameters in mice treated for 2 weeks with UAG (100 nmol/kg) or UAG_{6-13} (3,000 nmol/kg), with the last dose given 30 min prior to transient left coronary artery ligation (LCAL). (A) Body weight after 14 days of treatment. (B) Food and (C) Water consumption over the last 7 days of treatment initiation. (D) Glycemia, (E) Insulin levels and (F) NEFA plasma concentration were measured after 6 h of reperfusion following 30 min of myocardial ischemia. Data are mean ± SEM of *n* = 8 mice per treatment group and analyzed by one-way ANOVA followed by Student-Newman-Keuls' *post hoc* comparisons.

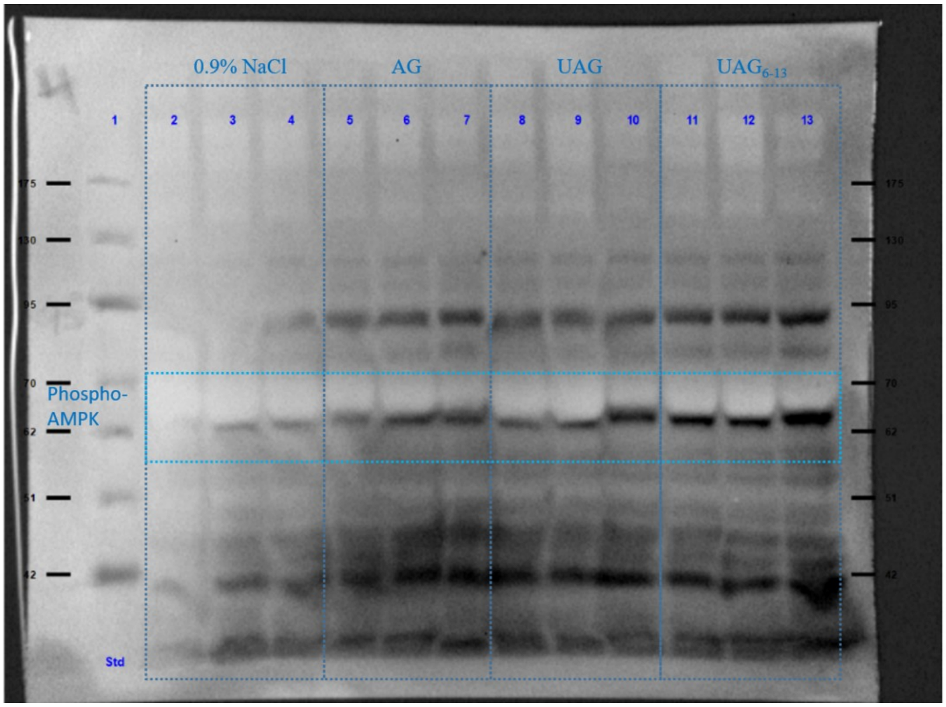

Supplemental Figure 2. Representative Western blot showing the levels of phospho-AMPK (62 kDa) in heart tissue cytosolic fraction.

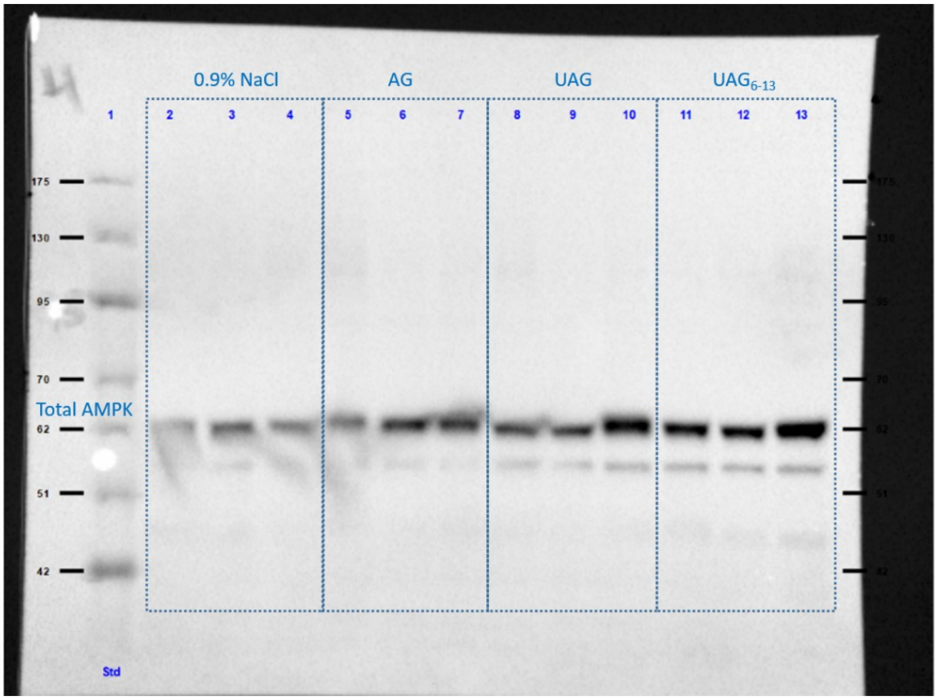

Supplemental Figure 3. Representative Western blot showing the levels of total-AMPK (62 kDa) after stripping of phospho-AMPK.

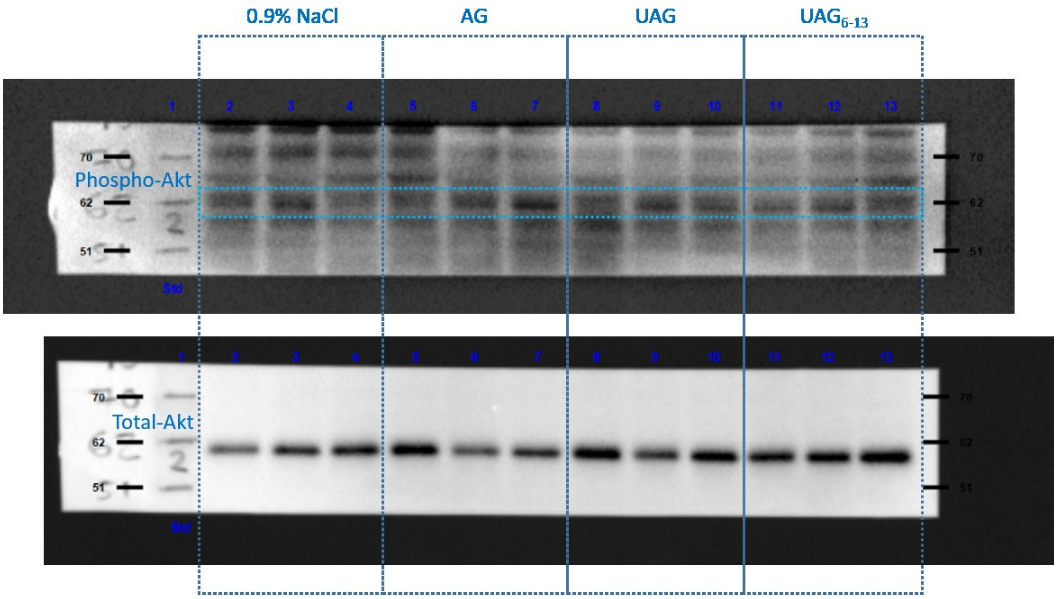

Supplemental Figure 4. Representative Western blot showing the levels of phospho-Akt (60 kDa) in heart tissue total homogenate and of total-Akt (60 kDa) after stripping of the membrane.

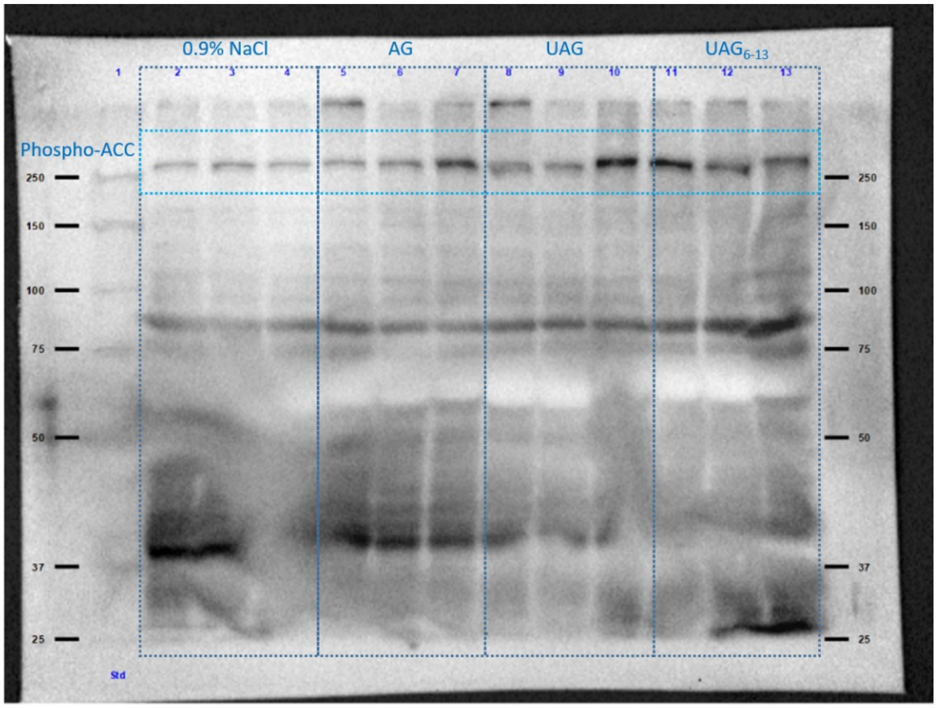

Supplemental Figure 5. Representative Western blot showing the levels of phospho-ACC (280 kDa) in heart tissue cytosolic fraction.

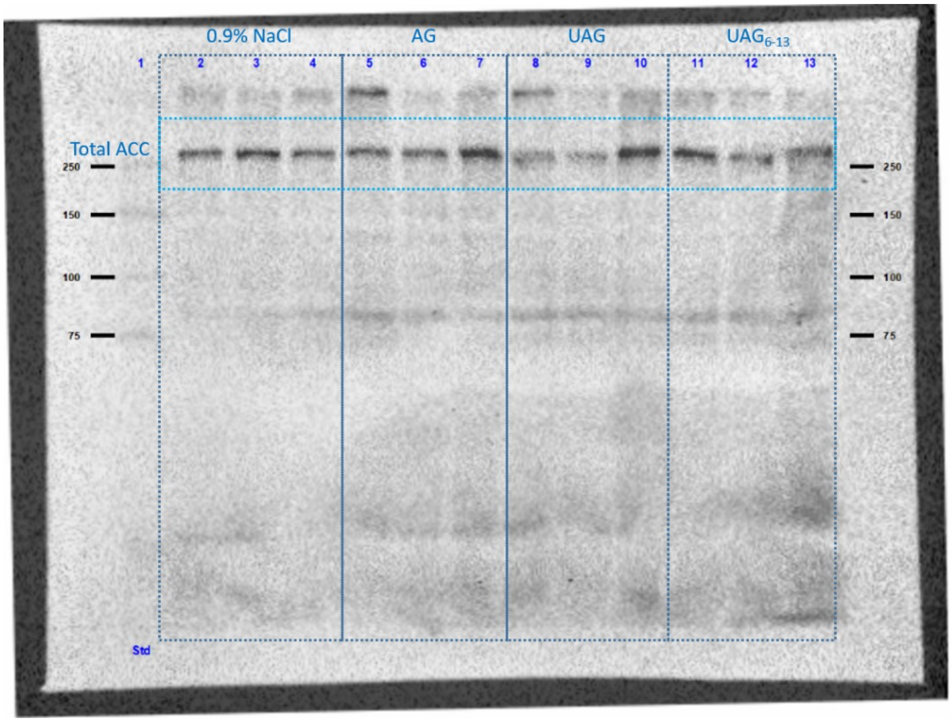

Supplemental Figure 6. Representative Western blot showing the levels of total-ACC (280 kDa) after stripping of phospho-ACC.

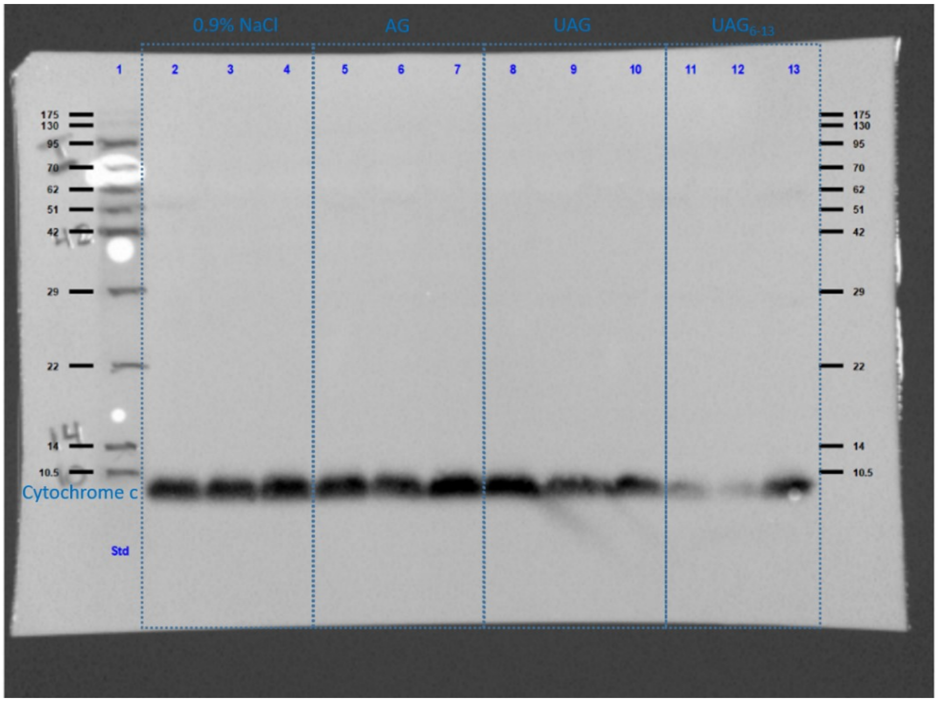

Supplemental Figure 7. Representative Western blot showing the levels of cytochrome c (8 kDa) in heart tissue cytosolic fraction.

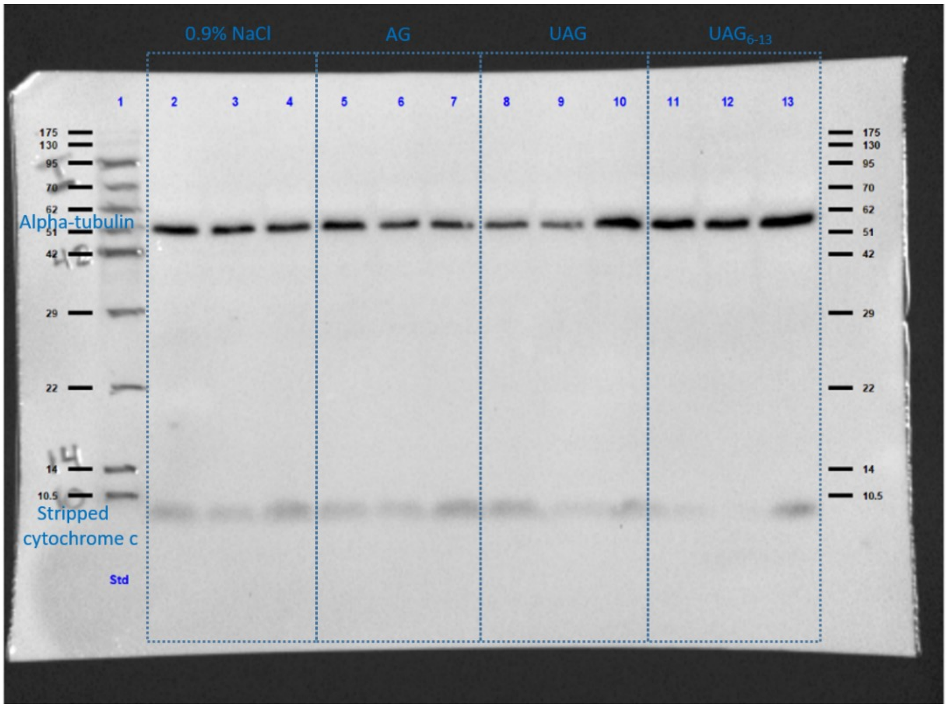

Supplemental Figure 8. Representative Western blot showing the levels of α-tubulin (50 kDa) after stripping of cytochrome c.

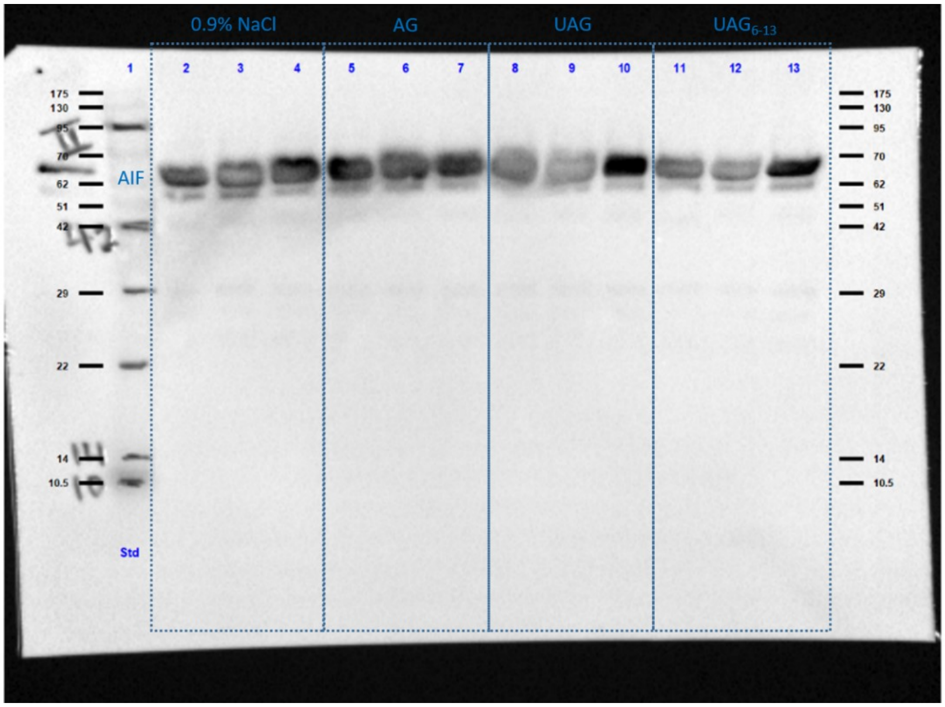

Supplemental Figure 9. Representative Western blot showing the levels of AIF (57 kDa) in heart tissue cytosolic fraction.

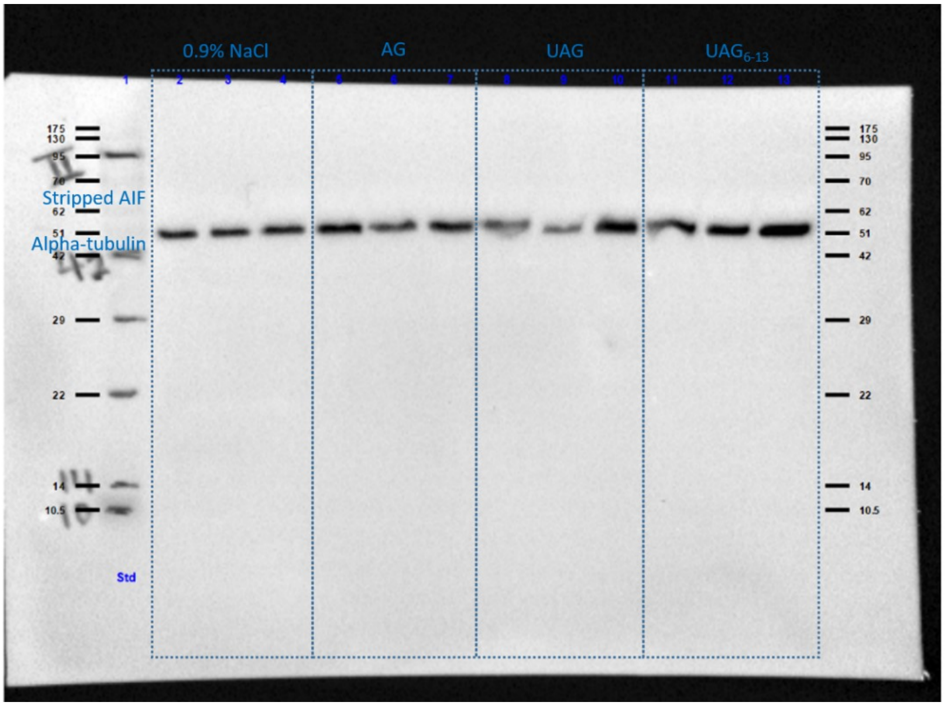

Supplemental Figure 10. Representative Western blot showing the levels of α-tubulin (50 kDa) after stripping of AIF.
